# Grazing Preference of Dairy Cows and Pasture Productivity for Different Cultivars of Perennial Ryegrass under Contrasting Managements

**DOI:** 10.3390/ani9050253

**Published:** 2019-05-20

**Authors:** M. Jordana Rivero, Oscar L. Balocchi, Fabián L. Neumann, Juan A. Siebald

**Affiliations:** 1 Departamento de Ciencias Agropecuarias y Acuícolas, Facultad de Recursos Naturales, Universidad Católica de Temuco, Campus Norte, Rudecindo Ortega 02950, Temuco 4780000, Chile; 2 Rothamsted Research, Sustainable Agriculture Sciences, North Wyke, Okehampton, Devon EX20 2SB, UK; 3 Facultad de Ciencias Agrarias, Instituto de Producción Animal, Universidad Austral de Chile, Campus Isla Teja, Valdivia 5090000, Chile; obalocch@uach.cl; 4 CIA-CENEREMA, Facultad de Ciencias Veterinarias, Universidad Austral de Chile, Campus Isla Teja, Valdivia 5090000, Chile; fabianneumann90@gmail.com; 5 Graduate School, Faculty of Agricultural Sciences, Universidad Austral de Chile, Campus Isla Teja, Valdivia 5090000, Chile; jasiebald@gmail.com

**Keywords:** high sugar grass, *Lolium perenne*, defoliation regime, nitrogen fertilisation, herbage mass, pasture yield, dry matter intake, pasture growth rate

## Abstract

**Simple Summary:**

Increasing soluble sugars in pasture species can lead to an improved nutrient use efficiency in the rumen and a greater digestibility of forage, which in turn might increase pasture intake. However, this improvement in nutritional value must not be at the expense of pasture productivity; the amount of nutrients harvested is a relevant factor in ruminant grazing systems’ efficiency. Therefore, we tested four different cultivars of perennial ryegrass (two selected for greater soluble sugar content and two standard cultivars) submitted to two contrasting managements (one aimed at improving sugar content and one with the opposite intended effect) for their effects on pasture productivity (by cutting herbage every time the plots reached the target number of leaves per tiller, i.e., two or three) and the grazing preference of dairy cows (six cows grazed for up to 5 hours, in an experimental area with three plots for each of the eight treatments) in spring, summer and autumn. We found that high sugar grasses had lower annual dry matter productivity and no preference was shown by cows, although the agronomic management aimed at reducing sugar concentration enhanced crude protein concentration and increased the herbage harvested (greater preference) in the three seasons, and the time spent grazing in autumn.

**Abstract:**

The objective of this study was to evaluate the pasture performance of different cultivars of perennial ryegrass, two “high sugar” and two standard cultivars, under two contrasting agronomic managements (aimed at either decreasing or increasing water soluble carbohydrates concentration), and their effects on the grazing preference of dairy cows. Eight treatments arising from the factorial combination of four cultivars and two managements were randomly applied to 31-m^2^ plots in three blocks. Pasture dry matter production and growth rate were measured for one year. Three grazing assessments were performed to establish the grazing preferences of six dairy cows in spring, summer and autumn. High sugar cultivars produced less dry matter per hectare than the standard cultivars. Cows consumed more grass and harvested a greater proportion of the pasture under the agronomic management aimed at decreasing sugar concentration, i.e., with a greater nitrogen fertilization rate and under a more frequent defoliation regime, which could be explained by the greater crude protein concentration achieved under this management. The results suggest that the genetic selection for greater levels of sugars was at the expense of herbage yield, and that cows preferred to graze herbage with a greater crude protein level instead of a greater sugar concentration.

## 1. Introduction

Perennial ryegrass (PRG), *Lolium perenne* L., is a grass distributed throughout the world of which there are a large number of cultivars, which differ mainly in ploidy level, heading date, type of endophyte fungus, disease resistance and water-soluble carbohydrates (WSC) content [1]. In the past, the selection of PRG cultivars has been based on increasing total yields, the seasonal distribution of yield, persistence, and resistance to diseases, with little focus on forage quality [2]. However, the improvement of nutritional quality has been relevant in recent decades to increase nutrients supply to grazing ruminants and to minimise their environmental impact [3]. An increased WSC concentration of PRG can be achieved by agronomic management, i.e., by decreasing the N fertilisation rate or decreasing the frequency of defoliation [4]. Through genetic selection, plant breeders have developed PRG cultivars with a greater concentration of WSC, known as “high sugar grasses” (HSG) [5]. Although it is desirable that the increase in WSC concentration achieved by breeding on HSG does not occur at the expense of dry matter (DM) yield [6], the results reported regarding the productivity of these cultivars have not been consistent [7,8,9,10,11].

WSC are completely digestible and have an important role in animal nutrition, since they are a primary source of readily available energy, necessary for efficient microbial fermentation in the rumen [12]. Increased microbial metabolism is a prerequisite for improving forage intake and nutrient utilisation, but this only occurs if the relations between WSC and crude protein (CP) are optimised [13,14]. Therefore, the use of PRG with a greater concentration of WSC allows a greater production of microbial protein and a greater flow of this protein to the duodenum, due to the better use of the protein, reducing nitrogen losses and decreasing the environmental impact [15]. Also, the greater concentration of WSC is expected to increase DM intake (DMI) and DM digestibility [16].

Regarding the effect of HSG on animal performance, the results reported did not always show any advantage for the use of this type of forage. There are in vivo studies [17] where DMI did not differ between forage from a HSG and a standard cultivar fed to dairy cows (11.6 vs. 10.7 kg DM day^−1^). However, the DM digestibility for the HSG diet was greater (0.71 vs. 0.64 g g^−1^ MS), which led to greater digestible DMI for that diet. Milk production of cows fed HSG forage (soiling) was greater (15.3 vs. 12.6 kg day^−1^). However, Taweel et al. [18] studied the effects of feeding lactating cows with PRG with a greater concentration of WSC on DMI, rumen function, milk production and composition. HSG cultivars had a greater WSC concentration than the standard cultivars and lower NDF and CP concentrations. The authors did not find a relationship between WSC concentration and DMI. However, Lee et al. [12] observed an increase in DMI when feeding confined steers for 21 days *ad libitum* with a diet based on PRG with a high WSC concentration. Differences in DMI intake between HS cultivars can be explained by the diet preference expressed when animals face no constrains during grazing [19]. Smit [20] reported that dairy cows consistently preferred perennial ryegrass cultivars with a high WSC concentration. This high preference may promote overgrazing and reduced persistence, but also may stimulate dairy cows to graze to low pasture residuals [21,22].

The objective of this study was to evaluate the pasture performance, in the first calendar year after establishment, of different cultivars of PRG, HSG and standard cultivars, under two contrasting agronomic management strategies, in terms of their effects on the concentrations of WSC and CP, and their effect on the grazing preference of dairy cows.

## 2. Materials and Methods

### 2.1. General Description of the Experiment

The study described here was part of an experiment reported previously by Rivero et al. [23] where the effects of cultivar and management on pasture quality were analysed for early spring and spring 2014, and summer and autumn 2015 in southern Chile (Valdivia, 39°47′ S, 72°12′ W). Briefly, treatments consisted of the factorial combination (repeated in three blocks) of four PRG cultivars, two high sugar cultivars (a diploid cultivar originated in New Zealand, 2nHSNZ, and a tetraploid cultivar originated in Europe, 4nHSEU) and two standard cultivars (one diploid cultivar, 2nSt, and one tetraploid cultivar, 4nSt) submitted to two contrasting agronomic managements to promote differences in WSC and CP concentrations (a low N—low frequency of defoliation regime (LNLF), consisting of defoliations at the stage of three leaves per tiller and with a N fertilisation rate equivalent to 83.3 kg N ha^−1^ year^−1^, and a high N—high frequency of defoliation regime (HNHF), consisting of defoliations at the stage of two leaves per tiller and with a N fertilisation rate equivalent to 250 kg N ha^−1^ year^−1^). Snip samples of these 31-m^2^ plots were taken during the first calendar year of establishment (sowing in March 2014) and analysed for WSC, CP, ME, NDF and ADF (cut at 5 cm above ground). Weather conditions during the grazing trial are presented in Rivero et al. [23].

### 2.2. Herbage Mass Production and Pasture Growth Rate

Cuttings were carried out over one year, from January 2015 to January 2016, each time the plots reached the target average number of leaves per tiller for each of the agronomic management groups (2 or 3 leaves). Herbage cutting was performed with a lawn mower (Husqvarna model 5521P) at a residue of 5 cm height. The edges of each plot were cut and the material discarded to eliminate the edge effect. Subsequently, the remaining area of the plot was measured to calculate the effective cutting surface. On the sampling dates when no grazing assessment was performed, the remaining forage of plots was harvested with the lawn mower and the herbage obtained from each plot was weighed, homogenised, and then a subsample was taken. This subsample was dried in a forced air oven at 60 °C for 72 h to estimate the percentage of DM to calculate the amount of DM harvested per plot and per hectare. The growth rate of the pasture from each plot was determined throughout the year (kg DM ha^−1^ day^−1^) with the DM herbage mass obtained from each cut and the days elapsed between cuts.

### 2.3. Grazing Preference

In October 2014, January 2015 and April 2015, both agronomic management groups, 12 HNHF plots and 12 LNLF plots, coincided in time regarding their target number of leaves per tiller, i.e., two and three, respectively. Then, on these sampling days, plots were grazed for between 3 and 5 h with six second-lactation Holstein–Frisian cows (average ± SEM: liveweight = 459 ± 7.2 kg and 79 ± 6.6 days in milk in October 2014). Animals had access to graze all the 24 plots simultaneously in order to establish the grazing preference using the technique of adjacent monoculture plots [24]. Water was available in the experimental area.

Before the animals entered the experimental area, and after removing the edges of each plot (83 cm width), a cut of a strip of each plot was collected with a lawn mower to determine the DM herbage mass (HM) prior to grazing. Once the grazing assessment was finished, cows were removed from the plots and the remaining herbage was harvested from the plots at a 5 cm residual height to determine DM HM post-grazing. Then, the herbage consumed by the cows was calculated for each plot and per ha. The determination of the DM percentage pre- and post-grazing was made following the same procedures as described for herbage mass production.

The grazing assessment was conducted from 8:00 a.m. (after morning milking) and for 5, 3 and 5 h for the grazing assessments carried out in October 2014, January 2015 and April 2015, respectively, to allow a residue greater than 5 cm in the plots. This ensured herbage allowance was not limiting herbage intake and allowed the determination of DM removed by grazing. The activity of the cows was observed every 10 min by one person to record if they were grazing and on which of the 24 plots. Total grazing time during each grazing assessment (spring, summer and autumn) was calculated by summing all the observations performed every 10 min, across all the cows and plots that were recorded as “grazing”. The percentage of grazing time for each of the 24 plots was calculated considering its accumulated grazing time (summing all the “grazing” behaviours for all the cows that grazed that plot) as a proportion of the total grazing time. These per plot values were used for the statistical analysis. Finally, to ease the presentation and interpretation of results, the percentage of time grazing for each group (each of the four cultivars for the “cultivar” factor and each of the two agronomic managements for the “management” factor) was calculated by multiplying the average percentage of grazing time per plot by the number of plots for each group (6 for each cultivar and 12 for each management).

### 2.4. Statistical Analysis

A complete randomised block design was used. For total annual DM herbage production, a factorial arrangement of treatments (four cultivars by two agronomic managements) was considered. Therefore, a two-way analysis of variance (ANOVA) was performed with the accumulated DM production measured throughout a complete year.

The statistical model used for the two-way ANOVA was as follows:

Y_hij_ = µ + b_h_ + α_i_ + β_j_ + (αβ)_ij_ + ε_hij_ (1)

where µ is the general mean, b is block effect, α and β are cultivar and management, respectively, followed by their interaction and the random error.

On the other hand, a one-way ANOVA was performed within each agronomic management to determine the effects of the cultivars on pasture growth rate, followed by a Tukey test (*p* = 0.05) for comparing means in those cases of significant differences.

A two-way ANOVA was performed for each grazing assessment (spring, summer and autumn) to determine the effects of the treatments on grazing preference. Additionally, analysis of covariance was performed to test whether the pre-grazing herbage mass affected either post-grazing residue, DM consumption, the propotion of forage harvested or the percentage of grazing time (covariate: pre-grazing HM). In cases of significant differences, a Least Significant Difference test (*p* = 0.05) was performed for comparing means. The Genstat^®^ 18th (©VSN International Ltd., Hemel Hempstead, UK) statistical system was used for all the analysis.

## 3. Results

### 3.1. Herbage Production

The annual herbage production of the treatments is shown in Table 1. Significant differences were found in annual DM production between cultivars (*p* < 0.05), with 4nSt showing the highest yield, although it did not differ from the other standard cultivar (2nSt). The HSG cultivars presented lower DM yields compared with the standard cultivars, although they did not differ statistically from the 2nSt cultivar. Regarding the effect of agronomic management, the HNHF management produced 18% more DM per annum than the LNLF management (*p* < 0.0001).

### 3.2. Pasture Growth Rate

The pasture growth rates of cultivars under HNHF management are shown in Table 2. Differences were only found in two cuts: those of May 26th (autumn) and July 20th (beginning of winter), when the cultivar 4nSt presented the highest growth rate.

The pasture growth rates of cultivars under LNLF management are shown in Table 3. Growth rates differed in the cuts of March 6th (end of summer), when the highest growth rate corresponded to the cultivar 4nSt, and October 29th (spring), when the highest growth rate corresponded to the cultivar 4nHSEU.

### 3.3. Summary of the Nutritional Quality of Forage for the Three Grazing Assessments

As reported by Rivero et al. [23], for the sampling periods analysed in the current study, WSC concentration only differed among cultivars in autumn when the 4nHSEU had a greater sugar concentration than the remaining cultivars (266 g/kg DM for 4nHSEU vs. 211 g/kg DM as an average of the other three cultivars), whilst the management only affected WSC in summer when the LNLF management promoted a greater WSC concentration (293 vs. 209 g/kg DM). Besides, CP concentration varied among cultivars, with 4nHSEU containing the greatest WSC levels (230 g/kg DM), 2nSt the lowest values (204 g/kg DM), and the remaining two cultivars containing intermediate values (218 g/kg DM on average). Moreover, CP concentration was greater for the HNHF management during the three sampling points reported here (spring 2014, summer 2015 and autumn 2015), containing on average 37% more CP than the LNLF management plots (see Table 1 and Table 2 of Rivero et al. [23]).

Rivero et al. [23] reported that ME content did not vary among cultivars nor between managements for any of the sampling periods (see Table 1 of Rivero et al. [23]). In general, diploid cultivars had greater NDF content in spring and autumn (average for cultivars 386 and 384 g/kg DM, respectively) compared with the tetraploid cultivars (average for cultivars 364 and 364 g/kg DM, respectively), with no difference in summer. However, the NDF concentration of pasture from both management groups did not differ in the period reported here. On the other hand, ADF concentration was the highest in spring for the 2nSt cultivar (241 g/kg DM) compared with the remaining three cultivars (average 224 g/kg DM). In summer, the 2nSt and 4nHSEU cultivars had the lowest values (216 g/kg DM on average), whilst in autumn the 4nHSEU cultivar had the lowest ADF concentration (206 g/kg DM) compared with the remaining cultivars (221 g/kg DM on average). However, ADF concentration only varied between managements in autumn when the HNHF management presented the lowest values (209 vs. 225 g/kg DM) (see Table 1 and Table 3 of Rivero et al. [23]).

### 3.4. Grazing Preference

Table 4 shows the results for grazing preference from the grazing assessments carried out in spring 2014, and summer and autumn 2015. Cultivar only affected the grazing variables in autumn, when the pre- and post-grazing HM was the lowest for the cultivar 4nHSEU (*p* < 0.05), but no differences were observed in DM consumption (after adjusting for the covariate pre-grazing HM), proportion harvested or the percentage of grazing time. Regarding agronomic management, pre- and post-grazing herbage mass differed in spring and summer, when the LNLF management promoted greater values for both outcomes (*p* < 0.05). Except for pre-grazing HM in autumn (*p* = 0.299), all the response variables related with forage consumption (HM, DMI and proportion harvested) were affected by management (*p* < 0.05); pre- and post-grazing HM was greater, DMI was lower and the proportion harvested was lower in the LNLF management compared with the HNHF. The interaction between cultivar and management observed in pre-grazing HM in spring 2014 is explained by the fact that under the HNHF management the HSG cultivars had the greater HM values (1053 vs. 862 kg DM ha^−1^), while under the LNLF management these cultivars had the lower HM values (1138 vs. 1439 kg DM ha^−1^), compared with the standard cultivars. Regarding the average percentage of time spent grazing in each plot, it only varied in autumn, when the HNHF plots showed a greater value compared with the LNLF plots, equivalent to 70 and 30 % of the total time spent grazing, respectively.

## 4. Discussion

### 4.1. Pasture Performance

The dry matter yields obtained in this study were low compared to the production potential of *Lolium perenne* L.; however, the agronomic management tested here might not have favoured the expression of the maximum DM yield potential. The annual production of PRG pasture in southern Chile can reach 9 t DM ha^−1^ in the edaphoclimatic system of the coastal mountain range, 12 t DM ha^−1^ in the central valley, 10 t DM ha^−1^ in the Andean foothills, and 5 DM ha^−1^ in the ñadis soils [25]. Other studies carried out in southern Chile report DM yields ranging from 7.7 t DM ha^−1^ year^−1^ [26] to between 12 and 15 t DM ha^−1^ year^−1^ [27]. Among other countries with similar climates, average DM yields of 12 and 13.4 t DM ha^-1^ year^-1^ were reported for two locations in New Zealand [10]. A study reported by Moscoso [28], carried out in the same area as the present study, obtained similar (even lower) DM yields, with averages of between 6.5 and 8.8 t DM ha^−1^ year^−1^. The low average DM yields of the present study might be explained by the nature of the realisation of the management and also, probably, to the summer drought of 2014/2015 that affected the area.

Due to the importance of increasing forage utilised for animal production, it is expected that the increase in the levels of WSC in HSG cultivars is not at the expense of a detriment in DM yield [6]. In this study, HSG cultivars had the lowest average DM yields in comparison with the standard cultivars. Studies carried out comparing the productivity of PRG cultivars show contrasting results. Halling et al. [7] conducted a study in nine sites in Europe and indicated that the total DM of Aberdart (HSG cultivar) was significantly lower than the standard cultivars in all sites except one site (in Wales). Similarly, O’Kiely et al. [29] compared the HSG cultivar Aberdart with Fennema (control) and found that the DM yields of Aberdart were lower in the four cuts analysed.

However, studies in New Zealand using a range of HSG cultivars derived from cultivars developed in the United Kingdom in a multi-site comparison showed that they did not consistently differ in performance from New Zealand controls [6]. Likewise, Bryant et al. [8] compared the cultivar Aberdart with two cultivars, the standard cultivar Samson and the standard cultivar tetraploid Quartet, and found no differences in the annual DM yield of the cultivars throughout two years of experimentation, with average yields of 9.3 and 10.3 DM ha^−1^ year^−1^ for the first and second year, respectively. Moscoso [28] did not find differences in the annual DM yield between HSG cultivars (Aberavon and Aberdart) and standard cultivars (Arrow and Jumbo) in a trial in the same area as the present study.

Regarding agronomic management, the HNHF management (aimed to reduce WSC concentration) presented a greater average DM production compared with the LNLF group (aimed to increase WSC concentration), producing 1.3 t DM more per year. This is explained by the fact that nitrogen fertilisation rate is one of the most important factors in determining yield, being even more relevant than the defoliation regime, i.e., number of leaves per tiller. Hopkins et al. [30] found that the DM production of a PRG pasture gradually increased up until a nitrogen fertilisation rate of 400 kg N ha^−1^ year^−1^, with no further increases observed above that dose up to 900 kg N ha^−1^ year^−1^. Besides, with the increase in nitrogen fertilisation rate there is an increase in tillering, due to the existence of a positive relationship between nitrogen and the production of cytokinins, compounds rich in nitrogen that are synthesised in the roots and would have a positive effect on tillering [31]. If this effect is added to the increase in the size and weight of the leaves in combination with the greater number of tillers, the increase in the production of herbage is explained to a great extent by increasing nitrogen fertilisation [32].

There is a clear pattern in the growth rate; in winter and summer seasons the growth rate decreased markedly for both managements, while in autumn and, for the most part, in spring the growth rate increased. This seasonality in growth rate and therefore in the production of dry matter is mainly due to the climate (precipitation and temperature). In spring the growth rate is maximal, while in the summer it decreases due to the water deficit, the high temperatures and the physiological maturity into which the plants enter. In winter, the growth rate is reduced due to low temperatures [33]. Teuber [34] found growth rates as low as 8 kg DM ha^−1^ day^−1^ in winter and as high as 58 kg DM ha^−1^ day^−1^ in spring in the central valley of Los Lagos Region (a neighbour region of the site used in this study), which is similar to the values found in the present study, with average values around 6 kg DM ha^−1^ day^−1^ in winter and 54 kg DM ha^−1^ day^−1^ in spring.

### 4.2. Grazing Preference

Preference can be calculated either as the proportion of time spent grazing each forage type, or as the proportion of intake derived from each forage type [35]. As stated by Smit et al. [36], animals had free choice to eat different sward types; therefore, the sward that was consumed most or grazed longest was considered as the most preferred sward.

Despite some seasonal differences in the nutritional quality of forage, there were no differences in the grazing preference of cows in relation to the cultivars tested. However, a greater consumption was observed in the HNHF management in all the grazing assessments, where the cows consumed on average 63% of the available forage above 5 cm. Smit [20] observed a clear difference in DMI in the first year of study between cultivars, but in the second year no differences were found. The author observed that a greater herbage intake was related to a greater mass of herbage and green leaf, a greater sward surface height, a lower infestation rate of the crown rust fungi and a lower lignin content in the herbage. Moreover, dairy cows very consistently preferred cultivars with a high WSC concentration, a high digestibility, a low cell wall concentration and a low ash concentration. Despite the differences observed in forage consumption between plots submitted to different grazing managements in this study, the time spent grazing only mirrored the proportion of forage removed in autumn, when the cows showed a partial preference (70% vs. 30%) for the forage from the LNLF.

The greater forage consumption from the pasture grazed at two leaves per tiller and with the greater fertilisation rate in this study (three times the N rate applied to the LNLF plots) is not explained by the ME or NDF content, since these nutritional components did not vary between managements for any of the grazing assessments. However, ADF content in autumn might contribute to partially explain the greater DM consumption in the HNHF plots since this nutritional component was lower in these plots [37,38]. Considering the main nutritional components intended to be manipulated in the current study, despite the fact that the WSC to CP ratio was greater for the LNLF management in all the grazing assessments (as intended), and despite the fact that the WSC in summer was greater for the LNLF management, the DM consumed seems to be directly related to the CP concentration of the swards, given that this nutritional component was greater for the HNHF management during the three grazing assessments. This preference for the forage containing more CP agrees with Poppi [37], who stated that it is common for ruminants to select a diet with greater digestibility, CP and minerals content. However, Mayland et al. [39] found that about one half of the variation in the preference score by cattle grazing a grass pasture was explained by a similar variation in the total non-structural carbohydrates (r = 0.71) and by the sum of monosaccharides and disaccharides (r = 0.67). Moreover, Smit et al. [36] found that dairy cows preferred cultivars with low ash and fibre concentrations and high WSC concentration and digestibility. Smit [20] concluded that in the selection process, the chemical quality of the herbage plays an important role, and the nutrient driving this selection by dairy cows in the current study seems to be the CP.

Teuber [40] indicates that grazing cattle define their preference for certain elements of the sward, distinguishing between plants and structures within the plant. The author also points out that this preference can be determined by previous experiences, fasting time, nutritional deficiencies or relative palatability. This is how they tend to prefer leaves over stems and generally more tender and green parts than older and senescent parts, and it is also observed that cattle reject the grass that has greater maturity and the sectors that contain dung.

## 5. Conclusions

Under the edaphoclimatic conditions of southern Chile, high sugar cultivars of perennial ryegrass produce less herbage per hectare annually than standard cultivars in the first calendar year after establishment. The growth rates of all the cultivars present a marked monthly variation, with the highest values in spring (October to December) and the lowest in winter (July to September), with the tetraploid cultivars tending to show some superiority in only a few cuts.

During the first spring, summer and autumn after sowing, cows consumed more grass and harvested a greater proportion of the perennial ryegrass pasture under the agronomic management with a greater nitrogen fertilisation rate and under a more frequent defoliation regime, which might be explained by the greater crude protein concentration achieved under this management, although other pasture characteristics not measured in the current study might also be influencing animal choice.

## Figures and Tables

**Table 1:** Annual dry matter (DM) yield of four *Lolium perenne* L. cultivars under two agronomic managements.

Factor	DM Annual Yield (kg DM ha^−1^ y^−1^)
Cultivar (C) ^1^	
2nSt	8578 ^a,b^
4nSt	8973 ^a^
2nHSNZ	7923 ^b^
4nHSEU	7927 ^b^
*p*	0.0333
Management (M) ^2^	
HNHF	9036
LNLF	7665
*p*	<0.0001
Interaction C × M	
*p*	0.8622

^a,b^ Values with different letters mean differing statistically (*p* < 0.05). ^1^ 2n: diploid; 4n: tetraploid; St: standard cultivar; HS: high sugar cultivar; NZ: origin New Zealand; EU: origin Europe. **^2^** High frequency of defoliation regime (HNHF): annual N fertilisation rate of 250 kg/ha and defoliated at the stage of two leaves per tiller; Low frequency of defoliation regime (LNLF): annual N fertilisation rate of 83.3 kg/ha and defoliated at the stage of three leaves per tiller.

**Table 2:** Growth rate (kg DM ha^−1^ d^−1^) of four *Lolium perenne* L. cultivars under a defoliation regime of two leaves per tiller and a fertilisation rate of 250 kg N ha^−1^ y^−1^ (from January 2015 to December 2015).

Cutting Date	Cultivar					
	2nSt	4nSt	2nHSNZ	4nHSEU	*p*	Average
Jan 7th	25.67	27.74	20.85	28.86	0.397	25.8
Feb 16th	6.41	6.16	6.31	5.78	0.892	6.2
Mar 6th	35.35	32.58	28.08	23.70	0.188	29.9
Mar 25th	41.90	35.47	43.81	30.13	0.399	37.8
Apr 23rd	45.01	49.93	38.34	33.81	0.086	41.8
May 26th	16.06 ^b^	22.83 ^a^	17.02 ^a,b^	13.12 ^b^	**0.005**	17.3
Jul 20th	5.56 ^b^	8.88 ^a^	6.46 ^a,b^	4.82 ^b^	**0.008**	6.4
Aug 27th	8.47	10.49	9.22	8.19	0.600	9.1
Oct 1st	21.16	26.21	23.00	27.20	0.226	24.4
Oct 29th	53.67	53.23	47.04	62.47	0.063	54.1
Nov 23rd	43.75	44.90	40.61	44.12	0.885	43.3
Dec 22nd	31.97	26.86	26.57	31.54	0.489	29.2

2n: diploid; 4n: tetraploid; St: standard cultivar; HS: high sugar cultivar; NZ: origin New Zealand; EU: origin Europe. ^a,b^ Values with different letters and *p*-values in bold mean differing statistically (*p* < 0.05).

**Table 3:** Growth rate (kg DM ha^−1^ d^−1^) of four *Lolium perenne* L. cultivars under a defoliation regime of three leaves per tiller and a fertilisation rate of 83 kg N ha^−1^ y^−1^ (from January 2015 to January 2016).

Cutting Date	Cultivar					
	2nSt	4nSt	2nHSNZ	4nHSEU	*p*	Average
Jan 7th	19.43	28.29	21.64	25.16	0.1761	23.6
Mar 6th	10.85 ^a,b^	14.09 ^a^	12.06 ^a,b^	6.84 ^b^	**0.0408**	11.0
Apr 23rd	29.85	30.87	27.52	21.68	0.4173	27.5
Jul 20th	8.30	9.65	7.00	4.32	0.0819	7.3
Sep 17th	10.93	11.61	10.62	10.75	0.8780	11.0
Oct 29th	32.41 ^a,b^	26.74 ^b^	25.98 ^b^	35.13 ^a^	**0.0237**	30.1
Dec 4th	48.34	42.25	41.88	45.55	0.6892	44.5
Jan 11th	19.10	18.54	18.17	15.33	0.3353	17.8

2n: diploid; 4n: tetraploid; St: standard cultivar; HS: high sugar cultivar; NZ: origin New Zealand; EU: origin Europe. ^a,b^ Values with different letters and *p*-values in bold mean differing statistically (*p* < 0.05).

**Table 4:** Pre- and post-grazing herbage mass, apparent DMI, proportion of DM harvested and the percentage of grazing time for the pastures of four *Lolium perenne* L. cultivars under two agronomic managements grazed by six dairy cows during three grazing assessments (spring, summer and autumn).

Factor	Grazing Spring 2014					Grazing Summer 2015					Grazing Autumn 2015				
	Pre-G HM	Post-G HM	DMI	Prop DM	% Time	Pre-G HM	Post-G HM	DMI	Prop DM	% Time	Pre-G HM	Post-G HM	DMI	Prop DM	% Time
Covariate ^1^															
*p*-value	-	0.434	**<0.0001**	0.061	0.235	-	0.633	**<0.0001**	**<0.0001**	0.777	-	0.150	0.001	0.107	0.491
Cultivar ^2^															
2nSt (*n* = 6)	1175	598	533	0.49	21.9	855	377	453	0.52	16.4	1369 ^a,b^	858 ^a^	436	0.38	22.8
4nSt (*n* = 6)	1125	536	587	0.51	13.7	912	357	471	0.55	21.6	1465 ^a^	755 ^a,b^	565	0.46	24.1
2nHSNZ (*n* = 6)	1020	488	619	0.53	39.7	692	354	485	0.56	27.9	1216 ^a,b^	748 ^b^	504	0.40	20.3
4nHSEU (*n* = 6)	1172	530	601	0.55	24.7	870	360	470	0.55	33.4	1011 ^b^	523 ^c^	673	0.49	32.9
SEM	64.2	46.0	48.5	0.043	8.40	80.4	26.2	28.3	0.034	9.42	100.9	70.3	76.0	0.059	4.56
*p*-value	0.352	0.435	0.796	0.799	0.299	0.275	0.924	0.652	0.320	0.572	**0.032**	**0.029**	0.293	0.549	0.264
Management ^3^															
HNHF (*n* = 12)	957	389	709	0.58	43.8	722	276	561	0.68	49.2	1211	453	798	0.62	69.6
LNLF (*n* = 12)	1289	687	461	0.46	56.2	942	448	379	0.41	50.8	1319	989	291	0.25	30.4
SEM	45.4	32.5	54.8	0.030	11.88	56.9	18.5	23.7	0.029	13.32	71.3	49.7	49.4	0.042	6.36
*p*-value	**<0.0001**	**<0.0001**	**0.002**	**0.017**	0.476	**0.016**	**<0.0001**	**<0.001**	**<0.001**	0.932	0.299	**<0.0001**	**<0.0001**	**<0.0001**	**<0.0001**
Interaction															
*p*-value	**0.016**	0.363	0.577	0.261	0.688	0.902	0.973	0.968	0.767	0.179	0.926	0.095	0.111	0.328	0.264

G: grazing; HM: herbage mass (kg DM ha^−1^); DMI: dry matter intake (kg DM ha^−1^); Prop DM: proportion harvested; % time: percentage of grazing time per level of the factor (calculated as a percentage of the total time spent grazing by the six cows during the whole observation period across all the 24 plots). ^a,b,c^ Values with different letters and/or *p*-values in bold mean differing statistically (*p* < 0.05). ^1^ When the covariate pre-grazing herbage mass had a significant effect on the remaining response variables, the mean and *p*-values presented are the adjusted values. ^2^ 2n: diploid; 4n: tetraploid; St: standard cultivar; HS: high sugar cultivar; NZ: origin New Zealand; EU: origin Europe. ^3^ HNHF: annual N fertilisation rate of 250 kg/ha and defoliated at the stage of two leaves per tiller; LNLF: annual N fertilisation rate of 83.3 kg/ha and defoliated at the stage of three leaves per tiller.
